# Supernumerary lacrimal puncta: case series and review of the literature

**DOI:** 10.11604/pamj.2023.45.140.37326

**Published:** 2023-07-24

**Authors:** Omar Mahmoud Solyman, Hashem Abdulaziz Abu Serhan, Mohammad Ali Tahboub, Mokhtar Mohamed Ibrahim Abushanab, Hesham Ali Hashem, Amr Mohammed Aref, Ahmed Sobh Abo Obaia, Hesham Foad Kamel

**Affiliations:** 1 Department of Ophthalmology, Research Institute of Ophthalmology, Giza, Egypt,; 2 Department of Ophthalmology, Qassim University Medical City, Al-Qassim, Saudi Arabia,; 3 Department of Ophthalmology, Islamic Hospital, Amman, Jordan,; 4 Department of Ophthalmology, Massachusetts Eye and Ear Infirmary, Boston, Massachusetts, United States

**Keywords:** Supernumerary puncta, double lacrimal puncta, punctum duplication, accessory punctum, lacrimal drainage system, dry eye, epiphora

## Abstract

We report a case series of supernumerary puncta-canaliculi, a very rare congenital anomaly, and describe different clinical presentations and new treatment options. This is a retrospective chart review of patients diagnosed with supernumerary lacrimal puncta during the time between June 2015 and December 2021 at the Research Institute of Ophthalmology, Giza, Egypt. Four patients (two females and two males) with a mean presenting age of 54 ± 14 years had unilateral double puncta. Of those four patients, three had double puncta on the right lower eyelid whereas one had double puncta on the left upper and lower eyelid. In one of the three patients, the double puncta anomaly was an incidental finding, and the patient was asymptomatic. The other three patients had associated epiphora. All four patients were found to have patent double puncta with no mechanical obstruction. No surgical interventions were necessary for all four patients as one resolved after discontinuing the topical eye drops. Another patient resolved after the diagnostic probing of the puncta, and the third asymptomatic patient required no interventions. Epiphora in the fourth patient resolved with botulinum toxin injection in the lacrimal gland. Accessory lacrimal puncta can present in patients as an incidental asymptomatic finding or patients may present with epiphora. Patients who present with unilateral epiphora, dry eye, or canaliculitis should be carefully evaluated with a detailed slit-lamp examination using lid eversion to appreciate potentially easily missed supernumerary lacrimal puncta.

## Introduction

Supernumerary puncta are rare congenital defects of the lacrimal drainage system. The incidence is not completely known. As cited in Chignell *et al*. a Wicherkiewicz report stated that it may be seen in 1 in 60000 eyes [1]; another report by Bacskulin *et al*. stated that it may be more common than previously thought, with an incidence of 1 in 800-1000 eye cases [2]. The underlying mechanism for this anomaly is still undetermined, however, it has been suggested that there may be an irregularity during embryological development [1,3,4]. It may begin with an irregular budding of the ectodermal core, which invades the mesoderm of the lid and becomes what is known as the lacrimal puncta-canaliculi [1]. Double lacrimal puncta are mostly asymptomatic, however, epiphora, dry eyes, and canaliculitis have been reported to be associated with these anomalies [1,3,5-14]. These findings can be easily missed during a routine ocular examination; thus, careful slit lamp examination is highly recommended to detect these anomalies. In this case series, we report four different patients with accessory lacrimal puncta with different clinical presentations. Along with our case series, multiple other case series and reports were compiled in this paper to demonstrate the presence of supernumerary puncta in the literature; the goal is to compare and highlight the treatments and interventions that may be of benefit to symptomatic supernumerary puncta patients.

## Methods

This is a case series report, a descriptive study based on a retrospective chart review of patients diagnosed with supernumerary lacrimal puncta during the time between June 2015 and December 2021 at the Research Institute of Ophthalmology, Giza, Egypt. All patients diagnosed with supernumerary lacrimal puncta were included. Consent was waived and publication was approved by the research ethics committee of the Research Institute of Ophthalmology.

## Results

Four patients (two females and two males) with a mean presenting age of 54 ± 14 years had unilateral double puncta. Of those four patients, three had double puncta on the right lower eyelid whereas one had double puncta on the left upper and lower eyelid. In one of the four patients, the double puncta anomaly was an incidental finding, and the patient was asymptomatic. The other three patients had associated epiphora. All four patients were found to have patent double puncta with no mechanical obstruction. No surgical interventions were necessary for all four patients as one resolved after discontinuing the topical eye drops. Another patient resolved after the diagnostic probing of the puncta, and the third asymptomatic patient required no interventions. Epiphora in the fourth patient resolved with botulinum toxin injection in the lacrimal gland.

**Figure 1:**
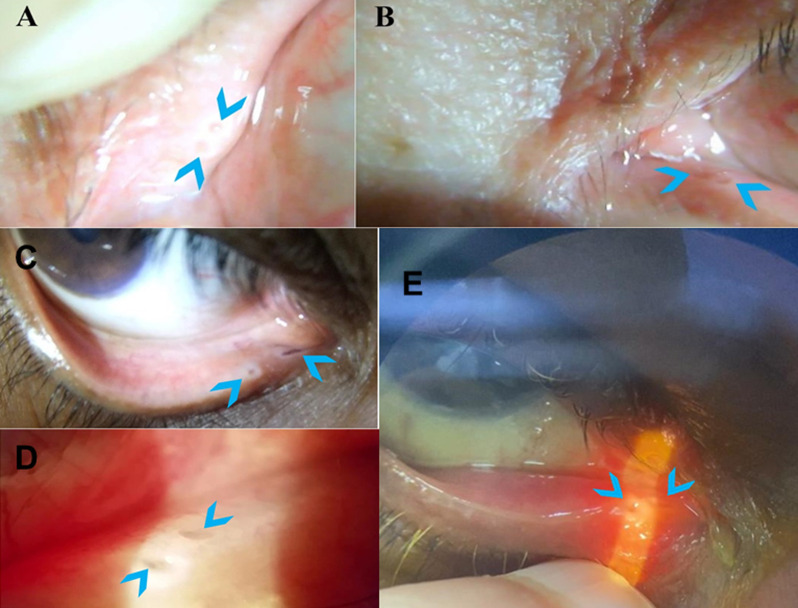
supernumerary puncta (blue arrows) (A, B) on slit lamp examination of patient-1 left upper and lower eyelids respectively; (C, D, E) supernumerary puncta on the lower right eyelid in patients 2, 3 and 4

**Case-1:** a 66-year-old female presented to the clinic with left eye tearing for two weeks. The patient had not undergone any ocular surgery except cataract surgery in the left eye 3 weeks prior to the presentation. The best corrected visual acuity (BCVA) was 20/20 right eye (OD) and left eye (OS). Intraocular pressure (IOP) was 13 mmHg OD and 14 mmHg OS. Slit lamp examination disclosed the presence of unilateral double punctum in both the left upper and lower eyelids (Figure 1A, B) along with increased fluorescein disappearance time in the left eye. Lacrimal drainage system irrigation demonstrated that each punctum was patent on both the left upper and lower eyelids. Simultaneous probing of each of the two puncta on both eyelids revealed an initial separate canaliculus for each punctum that joined to make a conjoint canaliculus about 5-6 mm in both eyelids. The excessive tearing was felt to be secondary to partial canalicular obstruction from the prolonged use of the postoperative topical antibiotic and corticosteroid eye drops post-cataract surgery. The patient was advised to discontinue the eye drops. She reported gradual improvement of tearing to complete resolution in about one month.

**Case-2:** a 39-year-old female with no known prior medical or ocular history or surgeries was referred to the oculoplastic service for evaluation of the right epiphora. The epiphora started gradually over the year prior to the presentation. The best corrected visual acuity was 20/20 OD and 20/25 OS. Intraocular pressure was 15 mmHg OD and OS. Slit lamp examination was remarkable for double puncta in the right lower eyelid (Figure 1C) along with a prolonged fluorescein dye disappearance test on the right side compared to the left side. Probing of the right lower eyelid showed patent puncta and canaliculi with no evidence of conjoint canaliculus for the double puncta on simultaneous probing. Irrigation of the lower punctum showed a patent nasolacrimal duct with partial regurgitation from the medial accessory punctum. The epiphora was likely a result of an incompetent lacrimal pump mechanism, however, the cause of the late presentation of the symptomatology could not be explained. The patient was consulted and offered interventions to relieve her symptoms including surgical occlusion of the medial accessory punctum to improve the lacrimal pump mechanism. The patient reported a subjective improvement of her right epiphora after the diagnostic probing and turned down other interventions.

**Case-3:** a 46-year-old male presented to the general eye clinic for evaluation of a stye on the lower eyelid of the right eye. He had a past ocular history of vernal keratoconjunctivitis as a child. The best corrected visual acuity was 20/25 OD and OS. Intraocular pressure was 16 mmHg OD and 19 mmHg OS. Slit lamp examination revealed double puncta on the right lower eyelid (Figure 1D). He was unaware of this anomaly and denied any other ocular symptoms including excessive tearing or dry eye. The patient´s dye disappearance test and Schirmer test were within normal limits for both eyes. No further workup was performed for this patient after the incidental finding was noted.

**Case-4:** a 67-year-old male patient with chronic sinusitis and an ocular history of essential blepharospasm for several years who newly established clinical care with our service complained also of tearing from the right eye for a few years. The patient reported that in summer, his right eye is usually full of tears that occasionally impair his vision and require dapping during his outdoor activity. During other months of the year, his vision is more frequently impaired with the tears that also overflow on his cheek and require more frequent dapping. Examination showed double lacrimal puncta in the right lower eyelid with a prolonged fluorescein disappearance test in the right compared to the left eye (Figure 1E). Probing and irrigation showed patency of both puncta with regurgitation of tears from the proximal punctum. A combination of a defective lacrimal pump and partial nasolacrimal duct obstruction was felt to be the cause of epiphora in the right eye. The patient was offered different treatment options but he opted to have a botulinum toxin injection in the lacrimal gland along with facial botulinum toxin injections for essential blepharospasm every three months. Four units of botulinum toxin- A were injected transconjunctivally into the palpebral lobe of the right lacrimal gland and the patient reported improvement of epiphora despite the persistence of the prolonged tear disappearance test.

## Discussion

The lacrimal punctum is an opening on the strong fibrous mound of the lacrimal papilla. The puncta are 0.2-0.3 mm in diameter with the inferior punctum and the superior punctum being 6.5 mm and 6.0 mm, respectively, away from the medial canthus [15]. Each respective punctum continues into the lacrimal canaliculus which passes through an approximately 2 mm vertical portion and an 8-10 mm horizontal portion [15]. The upper and lower canaliculi often merge to form a common canaliculus in about 90% of the population, which then empties into the sinus of Maier and through the one-way valve of Rosenmuller into the lacrimal sac [15]. The canaliculi have a mucosa that is composed of non-keratinized stratified squamous epithelium while the lacrimal sac is lined by pseudostratified epithelium [16]. And finally, the lacrimal fluid drains inferiorly from the lacrimal sac into the nasal cavity through the 12-18 mm long nasolacrimal duct [17].

Accessory lacrimal puncta and canaliculi are rare congenital anomalies that are underreported in the literature. We have compiled multiple case series and reports to exhibit trends and potential standardized treatments that may be of benefit to symptomatic double puncta patients (Table 1). In the table, there were a total of 51 patients with supernumerary puncta including the four patients in our case series [1,3,5-14]. There were a total of 27 female (53%) and 24 male (47%) patients. There were 17 supernumerary puncta patients that were asymptomatic in regard to the anomaly (33%), 30 patients with epiphora (59%), 2 patients with dry eye (4%), 2 patients with canaliculitis (4%), 1 patient with conjunctivitis (2%), and 1 patient with stage IV trachoma and chronic infection (2%). The total percentage added up to more than 100% due to some patients exhibiting multiple symptoms likely due to the presence of double puncta. And according to Chignell *et al*.[1] it was unlikely conjunctivitis and the chronic infection in the two patients from their case series were due to the double puncta. We had three patients presenting with epiphora and one patient was asymptomatic. Bair *et al*. and Chignell *et al*. suggested that the right lower lid is the most reported location for the occurrence of supernumerary puncta [1,6]. Thirty-nine patients out of the total 51 patients (76%) were found to have had right lower lid supernumerary puncta which is consistent with previous reports [1,3,5-14].

**Table 1:** summary of previous reports of supernumerary puncta

	# of pt with SP (total N=50; 27 F, 23 M)	x̄ age (years)	Eyelid	Symptoms	Associations	Interventions	Resolution of symptoms*
**Current case series**	4 (2 F, 1 M)	52	3 pt; RLL 1 pt; LLL+LUL	1 pt; asymptomatic; 3 pt; epiphora	None	1 pt; observation; 1 pt; cessation of prescribed eye drops; 1 pt; probing 1 pt; botulinum toxin	Yes, 3/3 pt
Satchi *et al*.	23 (12 F, 11 M)	54^a^	23 pt; RLL	5 pt; asymptomatic; 18 pt; epiphora	5/18 epiphora pts with NLDO while 6/18 had partial NLDO/functional epiphora; 1 pt; down syndrome; 2 pt; preauricular sinuses 6 children; NLDO, lacrimal fistula, lacrimal sac diverticulum, or absence of upper canaliculus	12 pt; DCR; 9 pt; observation; 2 pt; probing	Yes 2/2 pt; probing 11/12^b^ pt; DCR
Bair *et al*.	1 (F)	60	1 pt; RUL	1 pt; dry eye	None	1 pt; PP	Yes, 1/1 pt
Chignell *et al*.	6 (1 F, 5 M)	38	5 pt; RLL; 1 pt; RUL	5 pt; asymptomatic^c^; 1 pt; conjunctivitis^d^	1 pt; slit-like fistulas in canaliculi of the double punctum likely due to chronic infection	None^e^	N/A^e^
Galindo-Ferreiro *et al*.	1 (F)	60	1 pt; RLL	1 pt; dry eye	None	1 pt; PP	Yes, 1/1 pt
Yong *et al*.	1 (M)	59	1 pt; LLL	1 pt; canaliculitis	None	1 pt; oral antibiotics	Yes, 1/1 pt
Timlin *et al*.	5 (3 F, 2 M)	51	4 pt; LLL; 1 pt; RLL	5 pt; epiphora^f^	None	2 pt; DCR; 1 pt; punctoplasty; 1 pt; UPC+ probing^g^; 1 pt; observation^h^	Yes, 5/5 pt
Lacroix and Bitton	1 (F)	29	1 pt; RLL	1 pt; asymptomatic	None	None^i^	N/A
Chun and Yang	1 (F)	45	1 pt; RLL+RUL+ LLL+LUL	1 pt; bilateral epiphora	None	1 pt; bilateral CDCR	Yes, 1/1 pt
Azam *et al*.	1 (F)	18	1 pt; LLL^j^	1 pt; intermittent epiphora	None	None	No interventions
Opara *et al*.	1 (F)	19	1 pt; RLL^k^	1 pt; intermittent epiphora	None	1 pt; surgical removal of the supernumerary system	Yes, 1/1 pt
Ucar and Karadag	2 (2 F)	52	1 pt; RLL; 1 pt; LLL	1 pt; asymptomatic; 1 pt; epiphora	None	1 pt; DCR	Yes, 1/1 pt
Al Saleh *et al*.	4 (2 F, 2 M)	47	2 pt; RLL; 2 pt; LLL	4 pt; asymptomatic	2/4 pts; double punctum shared caniliculi^l^	None^e^	N/A^e^

F: female; M: male; pt: patient(s); RLL: right lower eyelid; LLL: left lower eyelid; LUL: left upper eyelid; RUL: right upper eyelid; NLDO: nasolacrimal duct obstruction; DCR: dacryocystorhinostomy; CDCR: conjunctivodacryocystorhinostomy; SP: supernumerary puncta; UPC: upper punctum canaliculotomy; PP: punctal plug

* In previously symptomatic patients; ^a^Median; ^b^from the 11/12 patients with a successful outcome, 2 required revised surgeries (1 repeat external DCR, 1 common canalicular membranectomy and silicone intubation); ^c^One patient had stage IV trachoma and watery/sticky in both eyes likely unrelated to the double puncta in her RUL and no interventions were done in regard to the double puncta; ^d^Doubtful the abnormal puncta/canaliculus contributed to the infection; ^e^No interventions done on patients in regard to their double puncta; ^f^1/5 patients also had associated canaliculitis; ^g^For the patient with associated epiphora and canaliculitis; ^h^Patient was treated conservatively for his OU blepharitis with no interventions and observation for puncta; ^i^Patient told to manually occlude puncta when using eyedrops in future; ^j^Triple puncta; ^k^Double puncta with one of the puncta on the inner caruncle; ^l^A rare phenomenon of unknown clinical significance.

Similarly, cases 2, 3, and 4 of our case series had the right lower eyelid affected. Our first case, however, had the left upper and lower eyelids affected. The supernumerary puncta most commonly occurs in doubles, however, the most recorded puncta number on an eyelid was four [2,18]. Our case series of four patients showed similar trends, with all four having only double puncta on any one eyelid. It was also consistent with the comprehensive table results which demonstrated the vast majority of patients presenting with double puncta on any one eyelid (98%) and only one patient out of the 51 (2%) with triple puncta in an eyelid that only had intermittent epiphora and no interventions performed [1,3,5-14]. Forty-six percent of patients (n=23) in the table were reported by Satchi *et al*. which is the largest case series to date of patients with supernumerary puncta [5]. In their series, the presence of double puncta was an incidental finding in only five patients, whereas the other 18 patients presented with unilateral epiphora on the same side as the double puncta [5].

Although accessory lacrimal puncta can be asymptomatic, a variety of symptoms have been attributed to the presence of double puncta including but not limited to dry eyes, epiphora, and canaliculitis [1,3,5-14] In this discussion, we will describe each of the three associated symptoms and their potential causes.

Dry eyes can be the sole manifestation of supernumerary puncta canaliculi. Several theories have been proposed including enhanced outflow of the supernumerary system leading to increased tear excretion in the affected eye, resulting in accelerated dryness [5,6]. Another theory was that the anatomical relationship between the site of origin of the accessory canaliculus and Horner´s muscle can determine the direction of tear flow within the accessory puncta and canaliculus, which can be the cause of both dry eye and epiphora [5]. Several case reports have described the insertion of punctal plugs as a successful intervention for dry eyes in the context of supernumerary puncta [6,7]. In addition to punctal plugs, thermal cautery had been described as an alternative line of management in cases of double puncta canaliculi [6].

Congenital supernumerary puncta can present as excessive unilateral tearing [3,5]. There are a few mechanisms that are suggested in the literature that may explain this presentation in many patients. One possible explanation for the excessive tearing is a reduced lacrimal pump function [3]. The lacrimal pump is important for drawing tears into the lacrimal sac through the contraction of Horner´s muscle. However, the presence of double puncta can affect the lacrimal pump´s mechanism which may result in excessive tearing [3]. Another possible explanation for the occurrence of epiphora in patients with a patent nasolacrimal duct is the reflux of tears through the accessory punctum; tears may flow back towards the path of least resistance, and in this case, it is likely back to the tear film rather than the nasolacrimal duct [3]. Nasolacrimal duct obstruction is a common condition seen in patients who have double puncta, and it is also a potential cause of epiphora presentation [5]. In fact, Satchi *et al*. found that 5 out of 18 patients who had symptoms of epiphora from their double puncta had complete nasolacrimal duct obstruction, while the remaining 13 patients had either partial nasolacrimal duct obstruction or functional epiphora [5].

The embryological development of the lacrimal drainage apparatus may play a significant role in these patients with epiphora along with supernumerary puncta and nasolacrimal obstruction. The lacrimal drainage system develops at 5-10 weeks gestational age, beginning with lacrimal sac development followed by simultaneous co-formation of the canaliculi and nasolacrimal duct from invaginated ectoderm [3,4]. This simultaneous co-development could signify that a developmental or environmental injury to the embryological ectoderm at this critical point in development can lead to a concurrent abnormality in the nasolacrimal duct development, thus, increasing its resistance to tearing flow and predisposing it to obstruction [3,4]. Another report described the development of congenital supernumerary puncta occurring due to irregular budding of the ectodermal core, which invades the mesoderm of the lid and becomes what is known as the lacrimal puncta-canaliculi [1]. An abnormality arising during development may explain the symptomatic epiphora presentation for a supernumerary puncta anomaly patient.

Lines of epiphora management and treatment ranged from no surgical interventions to dacryocystorhinostomy (DCR) or punctoplasty, reflecting the secondary possible mechanisms for each patient [1,3,5-14] Various case series have reported successful DCR procedures, including the largest report by Satchi *et al*. in which 11 out of 12 patients reported resolution or improved symptoms postoperatively [1]. Timlin *et al*. had a smaller sample size of DCR patients (n=2) but demonstrated similar success. They used a Munk scoring system to describe the before and after DCR severity of epiphora symptoms; the scores ranged from 0-4 where ´0´ corresponds to no epiphora and a score of ´4´ corresponds to epiphora requiring dabbing more than 10 times a day [3]. The Munk scores for both patients 4-8 weeks postoperatively went from ´4´ to ´1´ and ‘0´. In the same report, one patient underwent punctoplasty and reduced his epiphora from a Munk score of ´4´ to ´2´ eight weeks postoperative [3]. Chun and Yang also found success in a 45-year-old female patient with bilateral upper and lower double puncta and symptomatic epiphora; they performed bilateral DCR and found the patient to be symptom-free postoperatively [12]. Ucar and Karadag performed DCR on another epiphora double puncta patient and demonstrated a resolution of symptoms in the one-month follow-up postoperatively [13]. The improvement and resolution of symptoms in many of these patients suggest that DCR and punctoplasty may be reasonable interventions for symptomatic epiphora.

Case-4 in this series responded well to botulinum toxin-A injection into the palpebral lobe of the right lacrimal gland. Botulinum toxin injection into either the palpebral or the main lacrimal gland lobes has been described as a less invasive treatment option for patients with epiphora with canalicular obstruction [19,20]. This patient had essential blepharospasm for which he receives botulinum toxin injection 3-4 times a year. He was injected with 4 units of botulinum toxin in the palpebral lobe of the right lacrimal gland during a scheduled injection session for essential blepharospasm with the resolution of epiphora over 2-months of follow-up. To the best of our knowledge, this is the first description of botulinum toxin injection in the treatment of multiple lacrimal puncta-associated epiphoras. Although we had no cases of canaliculitis associated with the double punctum anomaly, it is important to discuss their association. Yong *et al*.described the first association of a 59-year-old male with double puncta and ipsilateral canaliculitis [8]. They have proposed that an abnormal embryological development may be the reason for such an association. The patient´s inner and outer punctum may have undergone different paths during development. The inner punctum underwent complete canalization maintaining its connection with the main epithelial cord. The outer punctum separated from the main epithelial cord, creating a cul-de-sac prone to pathology. This anomalous cul-de-sac became a nidus for infection due to the sequestration of tears and debris over time [8]. The resultant canaliculitis can be treated with oral antibiotics [8]. Another report by Timlin *et al*. described a patient with lower left eyelid double puncta and ipsilateral upper punctum canaliculitis [3]. They performed an upper punctum canaliculotomy to treat and resolve the infection and were not able to find upper canaliculus abnormalities, thus, they were unsure of the relation between the anomaly and the infection [3].

## Conclusion

Accessory lacrimal puncta can present in patients as an incidental asymptomatic finding or with a variety of symptoms. In our case series, we highlight four interesting cases of patients presenting either asymptomatically or with epiphora. Other case series have reported other symptoms such as canaliculitis. Patients who present with unilateral epiphora or dry eye should be carefully evaluated with a detailed slit-lamp examination using lid eversion to appreciate potentially easily missed supernumerary lacrimal puncta.

### What is known about this topic

- *Accessory lacrimal puncta and canaliculi are rare congenital anomalies that are underreported in the literature;*
- *Supernumerary puncta patients could be asymptomatic or presented with epiphora, dry eye, canaliculitis, conjunctivitis, trachoma, and chronic infection;*
- *The right lower lid is the most reported location for the occurrence of supernumerary puncta*.

### What this study adds

- *Double lacrimal puncta and canaliculi may predispose to canalicular obstruction medicamentosa;*
- *We described possible lines of epiphora management and treatment by giving recommendations about a stepwise management approach including the first use of botulinum toxin-A injection in the lacrimal for treatment of epiphora secondary to double lacrimal puncta and canaliculi;*
- *We report a rare case of double lacrimal puncta and canaliculi in both eyelids of the same eye*.

## References

[1] Chignell AH (1968). Double punctum and canaliculus. Am J Ophthalmol.

[2] Bacskulin J (1964). Double, triple and quadruple lacrimal puncta. Klin Monbl Augenheilkd.

[3] Timlin HM, Keane PA, Ezra DG (2019). Characterizing Congenital Double Punctum Anomalies: Clinical, Endoscopic, and Imaging Findings. Ophthalmic Plast Reconstr Surg.

[4] de la Cuadra-Blanco C, Peces-Peña MD, Jáñez-Escalada L, Mérida-Velasco JR (2006). Morphogenesis of the human excretory lacrimal system. J Anat.

[5] Satchi K, McNab AA (2010). Double lacrimal puncta: clinical presentation and potential mechanisms of epiphora. Ophthalmology.

[6] Bair PJ, Tsai YY, Lin JM (2004). Congenital reduplication of the lacrimal punctum and canaliculus in a patient with dry eye. Ophthalmic Surg Lasers Imaging.

[7] Galindo-Ferreiro A, AlGhafri L, Akaishi P, Galvez-Ruiz A, Galindo-Alonso J, Schellini S (2018). Punctal Plug as a Treatment Option for Dry Eye Associated with Congenital Supernumary Puncta. Middle East Afr J Ophthalmol.

[8] Yong KC, Kah TA, Annuar FH (2011). Canaliculitis in supernumerary puncta and canaliculi. Clin Pract.

[9] Lacroix Z, Bitton E (2015). Supernumerary punctum: an unusual case of seeing double. Clin Exp Optom.

[10] Afifa Azam SK, Samir Kumar Lal (2020). Supernumerary lacrimal puncta: A case report. Indian Journal of Clinical and Experimental Ophthalmology.

[11] Osorio RF (2017). Congenital supernumerary lacrimal puncta and canaliculus on the caruncle. J Clin Ophthal Optan.

[12] Chun JW, Yang SW (2013). A Case of a Congenital Lacrimal Outflow Dysgenesis with Supernumerary Lacrimal Puncta. J Korean Ophthalmol Soc.

[13] Ucar IC, Karadag R (2020). Double puncta canaliculi may exhibit different clinical presentations. Arq Bras Oftalmol.

[14] Al Saleh A, Vargas JM, Al Saleh AS (2021). Supernumerary lacrimal puncta: Case series. Saudi J Ophthalmol.

[15] Paulsen F, Garreis F, Schicht M, Bräuer L, Ali MJ, Sel S (2016). Anatomy and physiology of the nasolacrimal ducts. HNO.

[16] Kominami R, Yasutaka S, Taniguchi Y, Shinohara H (2000). Anatomy and histology of the lacrimal fluid drainage system. Okajimas Folia Anat Jpn.

[17] Tamboli DA, Harris MA, Hogg JP, Realini T, Sivak-Callcott JA (2011). Computed tomography dimensions of the lacrimal gland in normal Caucasian orbits. Ophthalmic Plast Reconstr Surg.

[18] Greeves RA (1914). Supernumerary Punctum Lachrymale and Canaliculus. Proc R Soc Med.

[19] Lee AG, Lee SH, Jang M, Lee SJ, Shin HJ (2021). Transconjunctival versus Transcutaneous Injection of Botulinum Toxin into the Lacrimal Gland to Reduce Lacrimal Production: A Randomized Controlled Trial. Toxins (Basel).

[20] Jeffers J, Lucarelli K, Akella S, Setabutr P, Wojno TH, Aakalu V (2022). Lacrimal gland botulinum toxin injection for epiphora management. Orbit.

